# *Arabidopsis *seedling flood-inoculation technique: a rapid and reliable assay for studying plant-bacterial interactions

**DOI:** 10.1186/1746-4811-7-32

**Published:** 2011-10-06

**Authors:** Yasuhiro Ishiga, Takako Ishiga, Srinivasa R Uppalapati, Kirankumar S Mysore

**Affiliations:** 1 Plant Biology Division, Samuel Roberts Noble Foundation, Ardmore, OK 73401, USA

## Abstract

**Background:**

The *Arabidopsis thaliana-Pseudomonas syringae *model pathosystem is one of the most widely used systems to understand the mechanisms of microbial pathogenesis and plant innate immunity. Several inoculation methods have been used to study plant-pathogen interactions in this model system. However, none of the methods reported to date are similar to those occurring in nature and amicable to large-scale mutant screens.

**Results:**

In this study, we developed a rapid and reliable seedling flood-inoculation method based on young *Arabidopsis *seedlings grown on MS medium. This method has several advantages over conventional soil-grown plant inoculation assays, including a shorter growth and incubation period, ease of inoculation and handling, uniform infection and disease development, requires less growth chamber space and is suitable for high-throughput screens. In this study we demonstrated the efficacy of the *Arabidopsis *seedling assay to study 1) the virulence factors of *P. syringae *pv. *tomato *DC3000, including type III protein secretion system (TTSS) and phytotoxin coronatine (COR); 2) the effector-triggered immunity; and 3) *Arabidopsis *mutants affected in salicylic acid (SA)- and pathogen-associated molecular pattern (PAMPs)-mediated pathways. Furthermore, we applied this technique to study nonhost resistance (NHR) responses in *Arabidopsis *using nonhost pathogens, such as *P. syringae *pv. *tabaci*, pv. *glycinea *and pv. *tomato *T1, and confirmed the functional role of FLAGELLIN-SENSING 2 (FLS2) in NHR.

**Conclusions:**

The *Arabidopsis *seedling flood-inoculation assay provides a rapid, efficient and economical method for studying *Arabidopsis-Pseudomonas *interactions with minimal growth chamber space and time. This assay could also provide an excellent system for investigating the virulence mechanisms of *P. syringae*. Using this method, we demonstrated that FLS2 plays a critical role in conferring NHR against nonhost pathovars of *P. syringae*, but not to *Xanthomonas campestris *pv. *vesicatoria*. This method is potentially ideal for high-throughput screening of both *Arabidopsis *and pathogen mutants.

## Background

One of the model pathosystems for the study of plant-pathogen interactions is *Arabidopsis thaliana-Pseudomonas syringae *interaction [1]. This model system has been widely used to understand a number of dynamic and complex molecular events in both resistance and susceptible interactions. In addition, *P. syringae *pvs. *tomato *and *maculicola *can infect and induce disease symptoms on *Arabidopsis. P. syringae *pv. *tomato *strain DC3000 (*Pst *DC3000), which causes bacterial speck disease of tomato, has been used as a model pathogen for investigating the molecular basis of plant-pathogen interactions because of its pathogenicity on *Arabidopsis *[1,2]. The whole genome sequence of *Pst *DC3000 revealed that it has over 200 virulence-related genes [3]. One of the major class of virulence factors includes effector proteins that are delivered into the host through a type III protein secretion system (TTSS) to suppress plant immune responses, and also to facilitate disease development [4]. *Pst *DC3000 also produces non-proteinaceous virulence effectors, including coronatine (COR), which are crucial for pathogenesis. However, the virulence function of a large number of potential virulence effectors encoded by the *Pst *DC3000 genome and their mode of action is still unknown.

Arabidopsis model system has been especially crucial in investigation of the plant defense mechanisms and signaling pathways underlying pathogen-associated molecular pattern (PAMP)-triggered immunity (PTI), effector-triggered immunity (ETI) and systemic acquired resistance [5-7]. The plant pattern recognition receptors, including FLAGELLIN-SENSING2 (FLS2), play an important role for FLS2-mediated PTI in the *Arabidopsis-Pst *DC3000 interactions. In addition to PTI, plants have evolved ETI via immune receptors (resistance proteins) to recognize corresponding avirulence effector proteins [6,8]. It has been shown that ETI and PTI use similar signaling pathways leading to defense responses [9,10]. Interestingly, pathogens have evolved virulence factors to target the hubs in plant immune system networks [11]. Therefore, to functionally dissect the dynamic interactions of plants with bacterial pathogens, there is a need for rapid, reliable pathogen assay that is suitable for high-throughput assays.

There are several reported methods to inoculate *Arabidopsis *with *P. syringae *including syringe pressure infiltration, vacuum infiltration, and spray- and dip-inoculation [1]. Syringe pressure infiltration is the most commonly used inoculation method, and the bacteria are forced into the apoplast using this method. However, in nature, *P. syringae *generally enters host tissues through natural openings such as stomata or wounds, and multiplies in the apoplast to cause disease [12]. In response to pathogen attack, *Arabidopsis *defense responses induce stomatal closure to limit the entry of bacteria after recognizing PAMPs from *P. syringae *[13]. When a COR-defective mutant was infiltrated into the apoplast by bypassing stomata-mediated defense, this mutant induced typical disease symptoms [13], suggesting that syringe pressure infiltration is not a suitable inoculation method for investigating the virulence mechanism of bacterial pathogens. Spray- or dip-inoculation methods have been used as a mimic for the natural infection process of *P. syringae*. However, these inoculation methods require high relative humidity to enable pathogens to enter and induce disease symptom development [1,14]. Spraying the abaxial leaf surfaces of the *Arabidopsis *rosette leaves without causing leaf damage is challenging, whereas the dip-inoculation of soil-grown plants is time consuming and requires the plants to be grown in pots with soil covered with nylon mesh. Moreover, the leaves inoculated with *P. syringae *using spray- and dip-inoculation methods do not show uniform disease symptoms because plant-pathogen interactions are often significantly affected by environmental factors and the developmental stage of the plants. Thus, the development of a reliable and robust inoculation method to study the interaction of *Arabidopsis *with *P. syringae *could reduce both time and space required.

Previously, we developed a simple tomato cotyledonary leaves-based assay to investigate *Pst *DC3000-tomato interactions and found that *Pst *DC3000 is a pathogen of tomato seedlings [15]. To establish an improved high-throughput assay to study the plant-bacterial interactions, in this study, we developed an improved, rapid and reliable seedling flood-inoculation method using *Arabidopsis*, a model plant that produces six to eight (true) rosette leaves within two-weeks, in standard Petri plates containing Phytagel supplemented with Murashige and Skoog (MS) salts. We further demonstrated that this method is suitable for the investigation of bacterial virulence mechanisms, plant nonhost resistance (NHR) and plant signaling pathways related to PTI and ETI.

## Results and Discussion

### *Arabidopsis *seedling flood-inoculation assay to study *P. syringae-Arabidopsis *interactions

To standardize the seedling assay and test whether *Pst *DC3000 multiplies and causes disease symptoms like in adult plants grown on soil, 2-week-old *Arabidopsis *seedlings (containing six to eight rosette leaves) grown on Phytagel plates were inoculated by flooding with a bacterial suspension until the plants were completely submerged in inoculum. The concentration of Phytagel and dryness of plates were critical for this assay. When the concentration of Phytagel was too low, the vitreous and wet plants were observed very often and were more sensitive to any pathogen inoculation. Seeds germinated on the plates made with 0.3% Phytagel produced seedlings that were the most suitable for the inoculation experiments.

**Figure 1:**
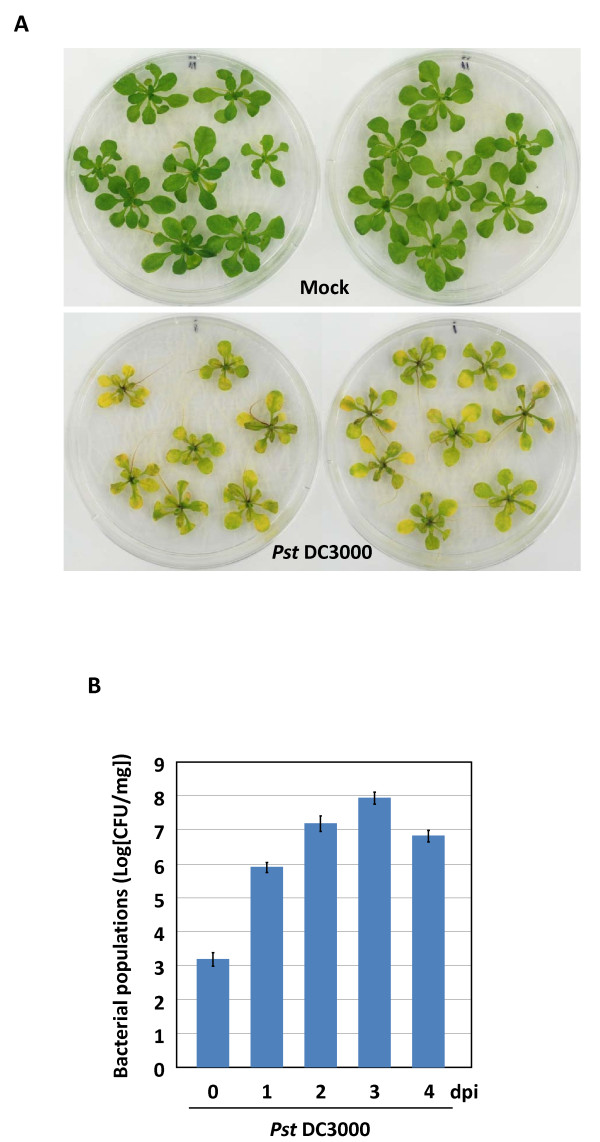
**A seedling flood-inoculation assay for the analysis of *Pseudomonas syringae *pv. *tomato *DC3000 (*Pst *DC3000) interactions with *Arabidopsis***. **A**. Disease phenotype of *Arabidopsis *seedlings flood-inoculated with a bacterial suspension of *Pst *DC3000 containing 0.025% Silwet L-77 at a concentration of 5 × 10^6 ^CFU/ml. Photograph was taken 3 dpi. Mock-inoculated seedlings were flood-inoculated with sterile distilled H_2_O containing 0.025% Silwet L-77. **B**. Bacterial populations of *Pst *DC3000 in *Arabidopsis*. Bacterial populations were quantified at 0, 1, 2, 3 and 4 dpi. Vertical bars indicate the standard errors for three independent experiments.

First, to study the effect of inoculum concentration on symptom development, *Arabidopsis *plants were flood-inoculated with three different concentrations of *Pst *DC3000 [1 × 10^8^, 2 × 10^7 ^and 5 × 10^6 ^colony-forming units (CFU)/ml)]. *Arabidopsis *seedlings exposed to 1 × 10^8 ^and 2 × 10^7 ^CFU/ml of bacteria showed severe disease symptoms including water-soaked lesions and chlorosis within 24-36 h and were dead by two to three days post-inoculation (dpi; data not shown). However, *Arabidopsis *plants inoculated with bacteria at 5 × 10^6 ^CFU/ml showed typical disease progression, showing chlorosis at 2 dpi and water-soaked lesions at 3 dpi (Figure 1A). However, at 5 dpi, the plants died due to severe disease (data not shown). The flood-inoculated *Arabidopsis *seedlings showed similar disease progression as that of soil-grown *Arabidopsis *plants inoculated with *Pst *DC3000 by vacuum infiltration at 1 × 10^6 ^CFU/ml bacterial concentration [1]. Thus, these results indicated that 5 × 10^6 ^CFU/ml bacterial concentration is suitable for further investigation of disease development.

In addition to the disease symptom development, the virulence of *Pst *DC3000 is generally investigated by measuring bacterial growth *in planta *[1]. In flood-inoculated *Arabidopsis *seedlings, *Pst *DC3000 multiplied approximately 1, 000-fold within the 24 hpi and reached 100, 000-fold at 3 dpi (Figure 1B). These results were similar to the bacterial growth curves observed in vacuum-infiltrated *Arabidopsis *mature leaves at 1 × 10^6 ^CFU/ml [1]. Furthermore, *Pst *DC3000 reached higher titer in seedling flood-inoculation assay compared to dip-inoculated leaves of soil-grown, 4-week-old *Arabidopsis *plants (Figure 1B; [14]). These results suggest that *Arabidopsis *seedling flood-inoculation assay is a reliable method to study *Pst *DC3000 disease progression and to evaluate *in planta *bacterial growth.

### *Arabidopsis *seedling flood-inoculation assay is suitable to study the virulence mutants of *Pseudomonas syringae*

**Figure 2:**
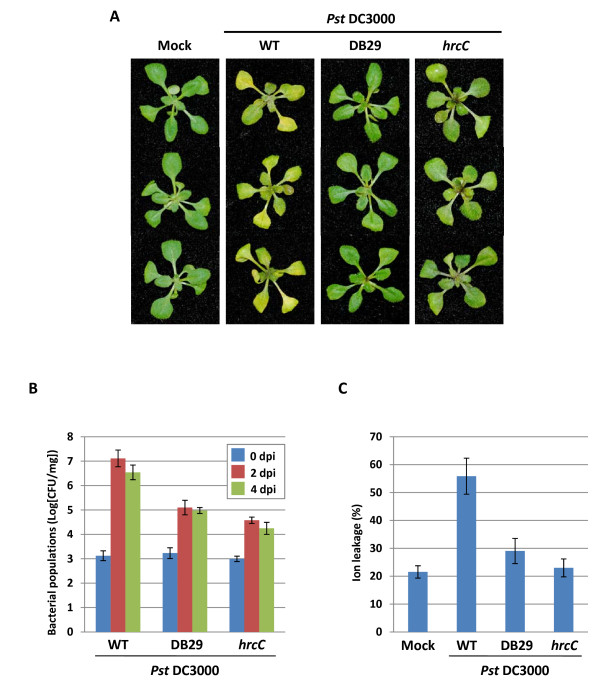
**A seedling flood-inoculation assay for the analysis of virulence factors in the pathogenicity of *Pseudomonas syringae *pv. *tomato *DC3000 (*Pst *DC3000) interactions with *Arabidopsis***. **A**. Disease phenotype of *Arabidopsis *seedlings flood-inoculated with a bacterial suspension of *Pst *DC3000, COR- mutant DB29 and type III secretion system (TTSS) mutant *hrcC *at a concentration of 5 × 10^6 ^CFU/ml. Mock-inoculated seedlings were flooded with sterile distilled H_2_O containing 0.025% Silwet L-77. Photographs were taken 3 dpi. **B**. Bacterial populations of *Pst *DC3000, DB29 and *hrcC *mutants in *Arabidopsis*. Bacterial populations were quantified at 0, 2 and 4 dpi. Vertical bars indicate the standard errors for three independent experiments. **C**. Ion leakage from *Arabidopsis *seedlings flooded with water (mock) or *Pst *DC3000, DB29 and *hrcC *mutants. The measurements were taken 3 dpi. Values show the percentage of total ions.

TTSS is a key virulence component of *P. syringae *because *hrp/hrc *mutants that block TTSS completely eliminate the virulence against susceptible *Arabidopsis *plants [16]. Furthermore, previous studies using COR-defective (COR^-^) mutants have demonstrated that COR enables *Pst *DC3000 to multiply and reach higher population densities *in planta*, and result in the development of larger lesions [12,14,17-21]. We used well characterized virulence mutants, including *hrcC *mutant [16] and DB29 as COR^-^d mutant [14] to study the utility of the *Arabidopsis *seedling flood-inoculation assay for investigating the virulence factors of *Pst *DC3000. *Pst *DC3000 caused typical water-soaked symptoms with severe chlorosis on *Arabidopsis *seedlings at 3 dpi (Figure 2A). However, water-soaked symptoms and chlorosis were not observed on DB29- and *hrcC*-inoculated seedlings, and they appeared healthy (Figure 2A). Consistent with disease development, the bacterial populations of DB29 and *hrcC *mutants were ~100-fold lower compared to *Pst *DC3000 (Figure 2B). Furthermore, a higher percentage of ion leakage (an indicator of disease-associated cell death) was observed in *Arabidopsis *seedlings inoculated with *Pst *DC3000 compared with those inoculated with DB29 or *hrcC *mutant (Figure 2C). These results indicate that both COR and TTSS have important roles in the bacterial multiplication, persistence and disease symptom development of *Pst *DC3000 in *Arabidopsis *seedlings and is consistent with the results obtained from soil-grown *Arabidopsis *plants [14,18,20]. These results further confirmed that the *Arabidopsis *seedling flood-inoculation assay is suitable for analyzing virulence mutants of *Pst *DC3000.

### *Arabidopsis *seedling flood-inoculation assay to study host signal pathways leading to disease development

*Arabidopsis coronatine insensitive 1 *(*coi1*) mutant demonstrated a role for jasmonate (JA)-mediated signaling pathway in defense against insects and necrotrophic pathogens [22,23]. *COI1 *encodes an F-box protein that functions as a receptor of COR and JA-isoleucine, and is considered a master regulator of the JA-mediated signaling pathway [22-27]. The *coi1 *mutant plants have been shown to be highly resistant to COR-producing *P. syringae*, including *Pst *DC3000 and *P. syringae *pv. *maculicola *ES4326 (*Psm *ES4326), with significant reduction of bacterial multiplication and disease symptom development [28,29]. Furthermore, we recently demonstrated a role for a suppressor of the G2 allele of skp1 (SGT1) in COR-induced chlorosis and *Pst *DC3000-induced disease development [30].

**Figure 3:**
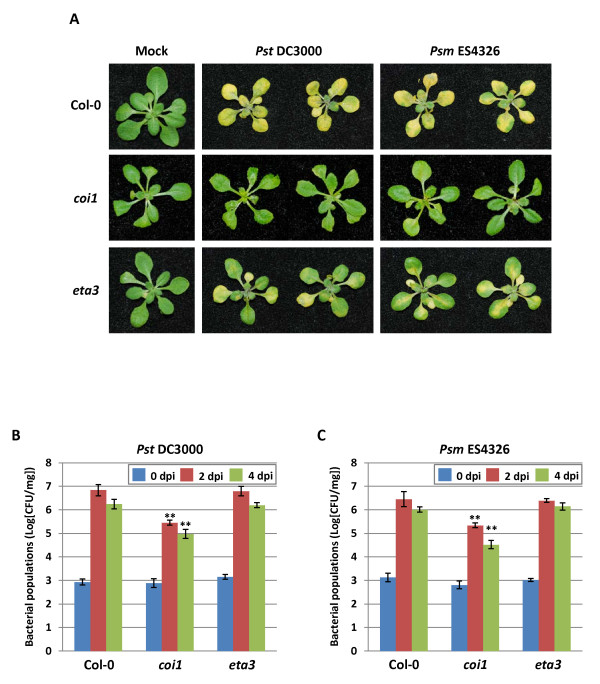
**A seedling flood-inoculation assay for the analysis of host signal pathways leading to disease development**. **A**. Disease phenotype of *Arabidopsis *seedlings flood-inoculated with pathogenic *Pseudomonas syringae *pv. *tomato *DC3000 (*Pst *DC3000) and *Pseudomonas syringae *pv. *maculicola *ES4326 (*Pm *ES4326) at a concentration of 5 × 10^6 ^CFU/ml. Mock-inoculated plants were flooded with sterile distilled H_2_O containing 0.025% Silwet L-77. Photographs were taken 3 dpi. (**B, C**) Bacterial populations of *Pst *DC3000 (**B**) and *Pm *ES4326 (**C**) in *Arabidopsis *were quantified at 0, 2 and 4 dpi. Vertical bars indicate the standard errors for three independent experiments. *Asterisks *indicate a significant difference from WT Col-0 using a *t*-test (** = *p *< 0.01).

To evaluate the utility of the *Arabidopsis *seedling flood-inoculation assay for studying host signaling pathways related to *Pst *DC3000- and *Psm *ES4326-induced disease susceptibility, we inoculated *Arabidopsis coi1 *and *sgt1b *(*eta3*) mutants along with the wild-type Col-0 with *Pst *DC3000 and *Psm *ES4326 using the flood-inoculation method. Both *Pst *DC3000 and *Psm *ES4326 caused typical water-soaked lesions with severe chlorosis on *Arabidopsis *wild-type seedlings at 3 dpi (Figure 3A). On the other hand, *coi1 *mutant seedlings inoculated with both pathogens appeared healthy without any water-soaked lesions or chlorosis (Figure 3A). In *coi1 *mutant seedlings, the bacterial populations of *Pst *DC3000 and *Psm *ES4326 were significantly lower compared to the wild-type Col-0 (Figures 3B and 3C). Consistent with our previous study [30], disease-associated water-soaked lesions and chlorosis were also significantly reduced in the *eta3 *mutant at 3 dpi with both the pathogens tested (Figure 3A). However, the bacterial populations of both pathogens were not different between wild-type and *eta3 *mutant (Figures 3B and 3C). Together, these results suggested that seedling flood-inoculation assay is suitable to further investigate the host signaling pathways leading to disease development in *Arabidopsis*.

### *Arabidopsis *seedling flood-inoculation assay to study effector-triggered immunity

**Figure 4:**
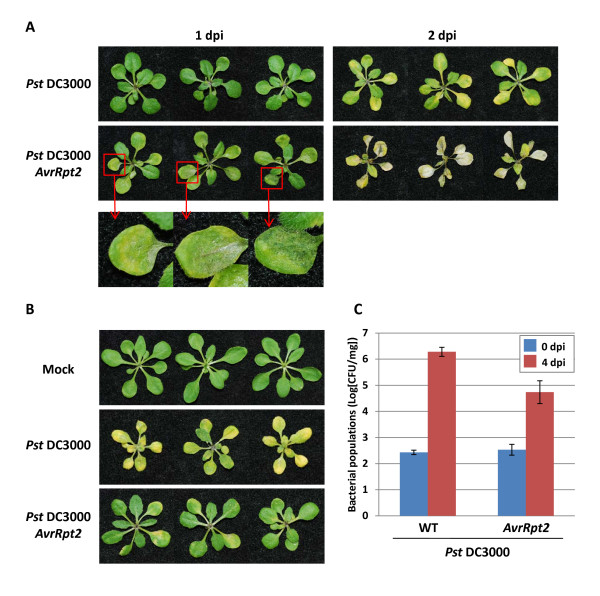
**Analysis of effector-triggered immunity using seedling-flood inoculation assays at high (5 × 10^6 ^CFU/ml) and low (1 × 10^5 ^CFU/ml) bacterial inoculums**. **A**. Response of *Arabidopsis *(Col-0) seedlings flood-inoculated with a bacterial suspension (5 × 10^6 ^CFU/ml) of *Pseudomonas syringae *pv. *tomato *DC3000 (*Pst *DC3000) and *Pst *DC3000 carrying *AvrRpt2 *(*Pst *DC3000 *AvrRpt2*) at 1 and 2 days post-inoculation. **B**. Response of *Arabidopsis *(Col-0) seedlings flood-inoculated with a low concentration (1 × 10^5 ^CFU/ml) bacterial suspension of *Pst *DC3000 and *Pst *DC3000 *AvrRpt2*. Photographs were taken at 4 dpi. **C**. Bacterial populations of *Pst *DC3000 and *Pst *DC3000 *AvrRpt2 *in *Arabidopsis *(Col-0) seedlings flood-inoculated with a low concentration (1 × 10^5 ^CFU/ml) of bacterial suspension. Bacterial populations were quantified at 4 dpi.

The *Arabidopsis-P. syringae *model system has been widely used to study ETI [1]. To evaluate the utility of the *Arabidopsis *seedling-flood inoculation assay for studying ETI, we inoculated *Arabidopsis *Col-0 that carries a resistance (*R*) gene *RPS2 *that can recognize AvrRpt2 with *Pst *DC3000 or *Pst *DC3000 carrying *AvrRpt2 *at high (5 × 10^6^ CFU/ml) and low (1 × 10^5^ CFU/ml) bacterial cell densities by flood inoculation. At high inoculum concentration, *Pst *DC3000 caused typical chlorosis on *Arabidopsis *wild-type (Col-0) seedlings at 2 dpi (Figure 4A). On the other hand, *Arabidopsis *seedlings inoculated with *Pst *DC3000 carrying *AvrRpt2 *showed HR as early as 1 dpi (Figure 4A inset) and complete cell death due to HR within 2 dpi (Figure 4A). At low inoculum concentration (1 × 10^5 ^CFU/ml), *Pst *DC3000 caused disease-associated water-soaked lesions and chlorosis at 4 dpi, whereas the seedlings inoculated with *Pst *DC3000 carrying *AvrRpt2 *appeared healthy (Figure 4B). Furthermore, the bacterial populations of *Pst *DC3000 carrying *AvrRpt2 *were significantly lower compared to those of *Pst *DC3000 (Figure 4C). Together, these results confirmed that *Arabidopsis *seedlings showed typical gene-for-gene mediated resistance responses and seedling-flood inoculation assay is suitable for analyzing ETI.

### *Arabidopsis *seedling flood-inoculation assay confirmed a role for *FLS2 *in nonhost bacterial resistance

**Figure 5:**
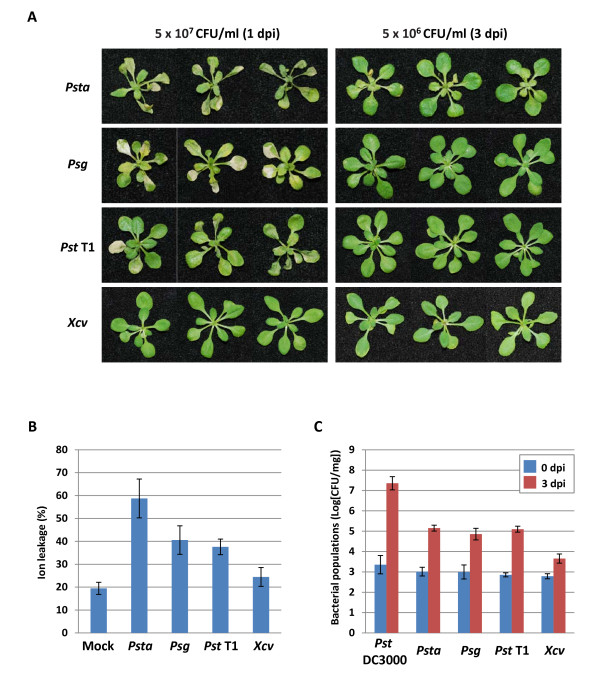
**Analysis of nonhost resistance responses of *Arabidopsis *using seedling flood-inoculation assay**. **A**. Phenotypes of *Arabidopsis *seedlings flood-inoculated with nonhost pathogens including *Pseudomonas syringae *pv. *tabaci *(*Psta*), *Pseudomonas syringae *pv. *glycinea *(*Psg*), *Pseudomonas syringae *pv. *tomato *T1 (*Pst *T1) and *Xanthomonas campestris pv. vesicatoria *(*Xcv*) at a concentration of 5 × 10^7 ^or 5 × 10^6 ^CFU/ml. Photographs were taken at 1 or 3 dpi. **B**. Ion leakage from *Arabidopsis *seedlings flooded with water (mock) or *Psta, Psg, Pst *T1 or *Xcv *at high bacterial density (5 × 10^7 ^CFU/ml) at 1 dpi. Bars show the percentage of total ions. **C**. Bacterial populations of *Pst *DC3000, *Psta, Psg, Pst *T1 or *Xcv *in *Arabidopsis *were quantified at 0 and 3 dpi. Vertical bars indicate the standard errors for three independent experiments.

After confirming the utility of the *Arabidopsis *seedling flood-inoculation method for identifying bacterial virulent mutants and plant mutants defective in disease signaling pathways, we applied the seedling flood-inoculation method to investigate the mechanisms of NHR in *Arabidopsis*. NHR is defined as a form of resistance exhibited by an entire plant species to a particular microbial pathogen and is the most common and durable form of resistance [31]. However, we know very little about various genes that regulate NHR [32]. Furthermore, the functional overlap between resistance mediated by ETI, PTI and NHR is not clear. We challenged *Arabidopsis *seedlings with nonhost bacterial pathogens including *P. syringae *pv. *tabaci *(*Psta*), pv. *glycinea *(*Psg*), pv. *tomato *T1 (*Pst *T1) and *Xanthomonas campestris *pv. *vesicatoria *(*Xcv*) at high (5 × 10^7^ CFU/ml) and low (5 × 10^6^ CFU/ml) bacterial cell densities by flood inoculation. At high inoculum concentration, *Psta, Psg *and *Pst *T1, but not *Xcv*, induced a hypersensitive response (HR) cell death within 24 hpi on *Arabidopsis *wild-type seedlings (Figure 5A). Furthermore, a higher percentage of ion leakage (an indicator of cell death) was observed in *Psta*-, *Psg*- and *Pst *T1-inoculated *Arabidopsis *seedlings when compared to *Xcv*- and mock-inoculated seedlings (Figure 5B).

At low inoculum concentrations (5 × 10^6 ^CFU/ml), none of the nonhost pathogens tested showed obvious symptoms on *Arabidopsis *plants (Figure 5A). In addition, the bacterial populations of *Psta, Psg, Pst *T1 and *Xcv *at low inoculum concentration were significantly lower compared to *Pst *DC3000 at 3 dpi (Figure 5C). Thus, these results indicate that *Arabidopsis *seedlings show typical NHR against *Psta, Psg, Pst *T1 and *Xcv*.

**Figure 6:**
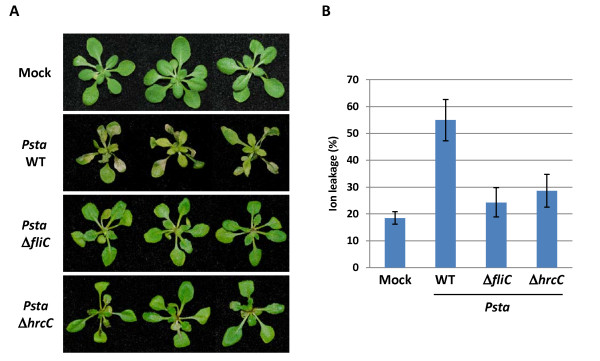
**Analysis of hypersensitive response (HR) cell death in *Arabidopsis *using seedling flood-inoculation assay**. **A**. HR of *Arabidopsis *seedlings flood-inoculated with *Pseudomonas syringae *pv. *tabaci *wild-type (*Psta*), *Psta *flagellin-defective mutant (*Psta *Δ*fliC*) and *Psta *type III secretion defective mutant (*Psta *Δ*hrcC*) at a concentration of 5 × 10^7 ^CFU/ml. Photographs were taken at 1 dpi. **B**. Ion leakage from *Arabidopsis *seedlings flooded with water (mock) or *Psta *(WT) or *Psta *Δ*fliC *or *Psta *Δ*hrcC *at 1 dpi. Bars show the percentage of total ions.

Interestingly, *Psta *induced stronger HR cell death in *Arabidopsis *than other nonhost pathogens tested (Figures 5A and 5B). It has been reported that nonhost plants recognize flagellin protein from *Psta *to induce HR cell death to limit bacterial growth via NHR [33-36]. Furthermore, TTSS was also shown to have a role in inducing HR cell death in *Arabidopsis-Psta *interactions [37]. To investigate whether flagellin or effector proteins can induce HR cell death using an *Arabidopsis *seedling flood-inoculation assay, we inoculated *Arabidopsis *wild-type seedlings with flagellin- and TTSS-defective mutants of *Psta *at 5 × 107 CFU/ml. *Psta *induced HR cell death within 24 hpi on *Arabidopsis *wild-type seedlings, whereas *Psta *Δ*fliC*- and Δ*hrcC*-inoculated *Arabidopsis *seedlings did not show any visible HR cell death (Figure 6A). Consistent with the cell death, a higher percentage of ion leakage was observed in *Psta*-inoculated *Arabidopsis *seedlings when compared to *Psta *Δ*fliC*- and Δ*hrcC*-inoculated seedlings (Figure 6B), suggesting that flagellin and TTSS are essential for the induction of HR cell death in *Arabidopsis-Psta *interactions.

**Figure 7:**
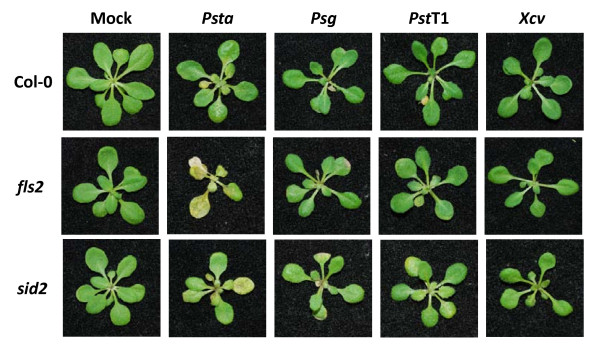
**Symptoms of *Arabidopsis *wild-type (Col-0), *fls2 *and *sid2 *seedlings flood-inoculated with nonhost pathogens *Pseudomonas syringae *pv. *tabaci *(*Psta*), *Pseudomonas syringae *pv. *glycinea *(*Psg*), *Pseudomonas syringae *pv. *tomato *T1 (*Pst *T1) and *Xanthomonas campestris pv. vesicatoria *(*Xcv*) at a concentration of 5 × 10^6 ^CFU/ml.** Photographs were taken 3 dpi.

**Figure 8:**
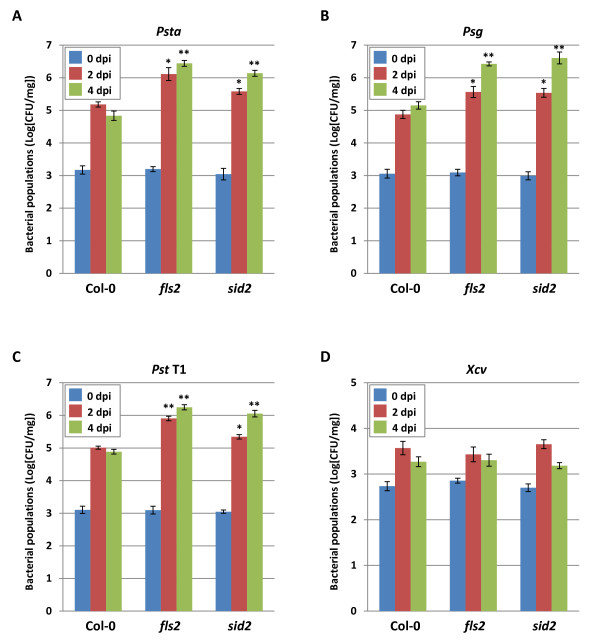
**Bacterial populations of nonhost pathogens *Pseudomonas syringae *pv. *tabaci *(*Psta*), *Pseudomonas syringae *pv. *glycinea *(*Psg*), *Pseudomonas syringae *pv. *tomato *T1 (*Pst *T1) and *Xanthomonas campestris pv. vesicatoria *(*Xcv*) in *Arabidopsis *wild-type (Col-0), *fls2 *and *sid2 *mutants. Bacterial populations of *Psta ***.(**A**), *Psg *(**B**), *Pst *T1 (**C**) and *Xcv *(**D**) were quantified at 0, 2 and 4 dpi. Vertical bars indicate the standard errors for three independent experiments. *Asterisks *indicate a significant difference from wild-type Col-0 in a *t*-test (* = *p *< 0.05, ** = *p *< 0.01).

Previous studies also demonstrated that flagellin-defective mutants of *Psta *evaded recognition by the nonhost plants and multiplied in tomato and *Arabidopsis *[33-35]. FLS2 was reported to have a role in NHR in *N. benthamiana *[38]. However, these studies have not convincingly demonstrated the precise role of flagellin perception as a component of NHR. Therefore, we inoculated *Arabidopsis *mutants defective in flagellin perception, *fls2*, and a SA biosynthetic mutant, *salicylic acid induction deficient 2 *(*sid2*), with nonhost pathogens *Psta, Psg, Pst *T1 and *Xcv *at 5 × 10^6 ^CFU/ml. Interestingly, only *Psta *induced disease-like symptoms associated with tissue chlorosis on *fls2 *and *sid2 *mutants (Figure 7). Furthermore, *fls2 *and *sid2 *supported higher *in planta *bacterial growth of nonhost pathogen *Psta *(Figure 8A), indicating the importance of flagellin-triggered immunity and the SA-mediated signaling pathway leading to NHR against *Psta*. It is important to note that the *Psta *flagellin-defective mutant caused disease-like symptoms in nonhost plants [33-35]. Taken together, these results suggest that *Psta *may have potential virulence mechanisms to cause disease once the NHR is compromised in these mutants. Interestingly, although *Psg *and *Pst *T1 failed to show any symptoms on *fls2 *and *sid2 *mutant seedlings, they supported higher levels of bacterial growth (Figures 8B, C), whereas *Xcv *failed to show any symptoms and did not multiply to higher levels in *fls2 *and *sid2 *mutant seedlings (Figure 8D). These results suggest that not all the nonhost pathogens have mechanisms to effectively deploy virulence factors (effectors or toxins) to cause disease even in the absence of the first layer of PTI mediated by *FLS2 *and NHR to *Xcv *may be mediated by the perception of PAMPs other than flagellin. It was reported that FLS2 did not detect all flagellin proteins among *Xanthomonas campestris *pv. *campestris *(*Xcc*) strains, and a Val-43/Asp polymorphism in flg22 region determined the PAMP activity of the *Xcc *flagellin protein [39]. It is interesting to note that the *Xcv *flagellin protein (GenBank accession numbers: CAJ23699.1 and YP_363753.1) represents a mutation in the Val-43 in the flg22 region (QQLSSGKRITSFAVDAAGGAIA) which may be undetectable by FLS2 in *Arabidopsis *[40].

## Conclusions

We have demonstrated that the *Arabidopsis *seedling flood-inoculation assay is a rapid and reliable assay for the study of interactions between *P. syringae *and *Arabidopsis*. In principle, we showed that this method should be suitable for investigating dynamic and complex molecular events, such as signaling pathways in both resistance and susceptible interactions. This assay could also provide an excellent system for investigating the virulence mechanisms of *P. syringae*. Due to high reliability and minimal space, time and budget requirements, this inoculation method is ideal for the high-throughput survey of *Arabidopsis *mutants altered in host-pathogen interactions. Furthermore, we also expect that this method will help to carry out pathogen mutant screens to elucidate the virulence mechanisms of phytopathogens that are pathogenic on *Arabidopsis *and especially beneficial for labs that have limited plant growth facilities.

## Methods

### Plant materials and growth conditions

*Arabidopsis thaliana *ecotype Colombia (Col-0) was used as a wild-type plant in this study. The male sterile *coi1-17 *line [41] was obtained from Dr. Barbara Kunkel (Washington University, St. Louis MO) and maintained as a heterozygous stock. The homozygous *coi1-17 *line was selected by growing the seeds from segregating lines on one-half Murashige and Skoog medium (MS) containing 10 μM methyl jasmonate (MeJA; Bedoukian Research Inc., Danbury, CT, U.S.A.) for seven days, and then transferring to one-half MS medium without MeJA. The *sid2-2 *(*eds16*) line [42,43] was obtained from Dr. Frederick Ausubel (Massachusetts General Hospital, Boston, MA). The *fls2 *line [36] was obtained from Dr. Yuki Ichinose (Okayama University, Okayama, Japan).

*Arabidopsis *seeds were sterilized using bleach. In brief, 100-200 seeds were incubated with 70% ethanol for 5 min in a microcentrifuge tube and then incubated with 20% (v/v) commercial bleach containing 6% sodium hypochlorite (Clorox Co., Oakland, CA) containing 0.1% Tween 20 (Sigma-Aldrich, St. Louis, MO, U.S.A.). After surface sterilization, seeds were washed with sterile distilled H_2_O at least four times and germinated on one-half strength MS medium containing Gamborg vitamins (PhytoTechnologies Laboratories, Shawnee Mission, KS, U.S.A.) solidified with 0.3% Phytagel (Sigma-Aldrich) in deep Petri plates (100 mm × 25 mm). The MS plates were dried overnight in the hood with closed lid before transferring the surface-sterilized seeds. The MS plates with seeds were kept for two days at 4°C to break the dormancy and were further incubated at 24°C with a light intensity of 150-200 μE m^-2 ^sec^-1 ^and a 12 h light/12 h dark photoperiod, and the seedlings, two weeks post-germination, were used for pathogen assays.

### Bacterial strains

*Pseudomonas syringae *pv. *tomato *DC3000 (*Pst *DC3000) [3] and *P. syringae *pv. *maculicola *ES4326 (*Psm *ES4326) [29] were used as pathogenic strains on *Arabidopsis*. The *hrcC *mutant defective in type III secretion [16] and a COR-defective mutant, DB29 [14], were used as virulence mutants of *Pst *DC3000. *Pst *DC3000 carrying *AvrRpt2 *[44] was used as an avirulent or incompatible pathogen to study ETI. Nonhost pathogens *P. syringae *pv. *tabaci *6605 (*Psta*) [45], pv. *glycinea *race 4 (*Psg*) [46], pv. *tomato *T1 (*Pst *T1) [47] and *Xanthomonas campestris *pv. *vesicatoria *(*Xcv*) [40] were used to study NHR. *Psta *Δ*fliC *mutant defective in flagellin [35] and the Δ*hrcC *mutant defective in type III secretion [48] were used to study HR cell death. *P. syringae *were grown at 28°C on mannitol-glutamate (MG) medium [49] containing appropriate antibiotics as needed in the following concentrations (μg ml^-1^): rifampicin, 50; kanamycin, 25; chloramphenicol, 25; and spectinomycin, 25, for 36-48 h. *Xcv *was grown at 28°C on Luria-Bertani (LB) media. Prior to inoculation, bacteria were suspended in sterile distilled H_2_O and bacterial cell densities (OD_600_) were measured using a Jenway 6320D spectrophotometer (Bibby Scientific Limited, Staffordshire, UK)

### Seedling flood-inoculation method

A flood-inoculation method that we have previously developed to infect the cotyledonary leaves of tomato [15] was modified to develop an *Arabidopsis *seedling flood-inoculation technique with reproducible disease symptoms. To perform uniform inoculation, 40 ml of bacterial suspension made in sterile distilled H_2_O containing 0.025% Silwet L-77 (OSI Specialties Inc., Danbury, CT, U.S.A.) was dispensed into the plate containing 2-week-old *Arabidopsis *seedlings, and the plates were incubated for 2-3 min at room temperature. After the bacterial suspension was removed by decantation, plates containing inoculated plants were sealed with 3 M Micropore 2.5 cm surgical tape (3 M, St. Paul, MN, U.S.A.) and incubated at 24°C with a light intensity of 150-200 μE m^-2 ^sec^-1 ^and a 12 h light/12 h dark photoperiod. Symptom development was observed at 1 and 3 dpi. In each experiment, 16 plants were evaluated, and each experiment was repeated at least three times.

To determine the bacterial growth in *Arabidopsis *leaves, we measured internal bacterial population at several time points (0, 1, 2, 3 and 4 dpi). Internal bacterial populations were evaluated from four biological replicates and each replicate represented a pooled sample of four independent seedlings from a single experiment grown in a single Petri-dish. Inoculated seedlings were collected by cutting the hypocotyls to separate the above agar parts (whole rosette) from the Phytagel plate, and the total weight of inoculated seedlings was measured. After measurement of the seedlings' weight, the seedlings were surface-sterilized with 5% H_2_O_2_ for 3 min. After washing three times with sterile distilled water, a pooled sample of four seedling were homogenized in 10 mL sterile distilled water using a mortar and pestle, and diluted samples were plated onto MG or LB medium containing the appropriate antibiotics. Two days after plating of diluted samples, the bacterial colony forming units (CFU) were counted using proper diluted samples. The CFU was normalized as CFU/mg using total weight of inoculated seedlings. Bacterial populations were evaluated in three independent experiments. 

### Detection of cell death

HR and disease-associated cell death were estimated by measuring ion leakage from five independent seedlings treated with water (mock) or inoculated with *P. syringae *and incubated for two days at 24°C with a light intensity of 150-200 μE m^-2 ^sec^-1 ^and a 12 h light/12 h dark photoperiod as described previously [50]. Inoculated seedlings (whole rosette) were collected by cutting the hypocotyls at the interface of the Phytagel plate and then gently agitated in 30 ml of distilled water for 3 h, and the leachates were measured using an ion conductivity meter (Orion555A, Thermo Fisher Scientific, Waltham, MA, U.S.A.). Plants were then autoclaved for 20 min to kill the cells and release total ions into the medium. Values relative to the whole ion content after autoclaving were used to express the percent ion leakage.

## List of abbreviations

TTSS: type III protein secretion system; COR: coronatine; SA: salicylic acid; PAMPs: pathogen-associated molecular patterns; FLS2: FLAGELLIN-SENSING 2; PTI: PAMP-triggered immunity; ETI: effector-triggered immunity; *Pst *DC3000: *Pseudomonas syringae *pv. *tomato *strain DC3000; MAMPs: microbe-associated molecular patterns; MS: Murashige and Skoog; CFU: colony-forming unit; dpi: days post-inoculation; JA: jasmonate; COI1: CORONATINE INSENSITIVE 1; *Psm *ES4326: *P. syringae *pv. *maculicola *ES4326; SGT1: suppressor of G2 allele of skp1; NHR: nonhost resistance; *Psta: Pseudomonas syringae *pv. *tabaci*; *Psg: Pseudomonas *pv. *glycinea*; *Pst *T1: *Pseudomonas *pv. *tomato *T1; *Xcv: Xanthomonas campestris *pv. *vesicatoria*; HR: hypersensitive response; SID2: SALICYLIC ACID INDUCTION DEFICIENT 2; MeJA: methyl jasmonate; MG: mannitol-glutamate; LB: Luria-Bertani.

## Competing interests

The authors declare that they have no competing interests.

## Authors' contributions

YI developed the seedling flood-inoculation technique, performed the experimental work and wrote a draft of the manuscript. TI performed the experimental work. SRU and KSM designed and coordinated the project and wrote the manuscript. All authors have read and approved the final manuscript.
